# Trigeminal activation patterns evoked by chemical stimulation of the dura mater in rats

**DOI:** 10.1186/s10194-020-01169-4

**Published:** 2020-08-15

**Authors:** Klaudia Flóra Laborc, Eleonóra Spekker, Zsuzsanna Bohár, Mónika Szűcs, Gábor Nagy-Grócz, Annamária Fejes-Szabó, László Vécsei, Árpád Párdutz

**Affiliations:** 1 Department of Neurology, Faculty of Medicine, Albert Szent-Györgyi Clinical Center, University of Szeged, Semmelweis u. 6, Szeged, H-6725 Hungary; 2 MTA-SZTE Neuroscience Research Group, Szeged, Hungary; 3 Department of Medical Physics and Informatics, Faculty of Medicine, Albert Szent-Györgyi Clinical Center, University of Szeged, Szeged, Hungary; 4 Faculty of Health Sciences and Social Studies, University of Szeged, Szeged, Hungary; 5 Interdisciplinary Excellence Center, Faculty of Medicine, University of Szeged, Szeged, Hungary

**Keywords:** Headache, Migraine, Trigeminal system, Animal model, Inflammatory soup, Complete Freund’s adjuvant, C-Fos

## Abstract

**Background:**

Although migraine is one of the most common primary headaches, its therapy is still limited in many cases. The use of animal models is crucial in the development of novel therapeutic strategies, but unfortunately, none of them show all aspects of the disease, therefore, there is a constant need for further improvement in this field. The application of inflammatory agents on the dura mater is a widely accepted method to mimic neurogenic inflammation in rodents, which plays a key role in the pathomechanism of migraine. Complete Freund’s Adjuvant (CFA), and a mixture of inflammatory mediators, called inflammatory soup (IS) are often used for this purpose.

**Methods:**

To examine the activation pattern that is caused by chemical stimulation of dura mater, we applied CFA or IS over the right parietal lobe. After 2 h and 4 h (CFA groups), or 2.5 h and 4 h (IS groups), animals were perfused, and c-Fos immunoreactive cells were counted in the caudal trigeminal nucleus. To explore every pitfall, we examined whether our surgical procedure (anesthetic drug, stereotaxic apparatus, local lidocaine) can alter the results under the same experimental settings. c-Fos labeled cells were counted in the second-order neuron area based on the somatotopic organization of the trigeminal nerve branches.

**Results:**

We could not find any difference between the CFA and physiological saline group neither 2 h, nor 4 h after dural stimulation. IS caused significant difference after both time points between IS treated and control group, and between treated (right) and control (left) side. Stereotaxic frame usage had a substantial effect on the obtained results.

**Conclusions:**

Counting c-Fos immunoreactive cells based on somatotopic organization of the trigeminal nerve helped to examine the effect of chemical stimulation of dura in a more specific way. As a result, the use of IS over the parietal lobe caused activation in the area of the ophthalmic nerve. To see this effect, the use of lidocaine anesthesia is indispensable.

In conclusion, application of IS on the dura mater induces short-term, more robust c-Fos activation than CFA, therefore it might offer a better approach to model acute migraine headache in rodents.

## Background

According to the Global Burden of Disease Study [1], migraine is the third highest cause of disability worldwide in both men and women. Although many specific drugs are available, the treatment of migraine is limited in many cases [2]. Its exact pathophysiology is still unknown, which makes drug development even more challenging. It is known that during the migraine attack the trigeminal system becomes sensitized and remains overactive, but there are several hypotheses about the initial cause without a clear answer [3]. Finding a reliable animal model would be crucial, though a model which shows all the aspects of the disease is still not available. Use of Complete Freund’s Adjuvant (CFA) or inflammatory soup (IS) on the dural surface is a proven useful method to cause trigeminal activation and sensitization in rats and these agents can stimulate neurogenic inflammation [4]. In this model, the activation and sensitization of the second-order sensory neurons occur between 2 and 4 h after application of IS similar to the cutaneous allodynia in migraine patients [5, 6].

The three sensory branches of trigeminal nerve supply the innervation of the dura mater [7, 8] and their somatotopic representation in the brainstem is already well described [9, 10]. However, the effect of chemical stimulation of the dura has never been studied before in regard to the representation of the nerve branches.

Expression of Fos protein is a well-known marker of activation in the trigeminovascular system [11]. In the chemical activation model, c-Fos expression might be induced by many stimuli (anesthetic drug, skin incision, ear bars of the stereotaxic apparatus, meningeal irritation, etc.) [12]. Knowing these effects would be essential for the proper analysis of the specific stimulus, to avoid pitfalls of the model. Thus, the aim of our study is to characterize the neuronal activation using two different inflammatory agents on the dura of rats and to determine the effects of surgery, and other altering factors on the expression of the Fos protein.

## Methods

### Animals

Forty-eight adult male Sprague-Dawley rats (weight 240–430 g) were used. The animals were raised and housed under standard laboratory conditions, light-dark cycle 12–12 h, regular rat chow and water ad libitum. The procedures used in our study were approved by the Committee of the Animal Research of University of Szeged (I-74-49/2017) and the Scientific Ethics Committee for Animal Research of the Protection of Animals Advisory Board (XI./1098/2018), and followed the guidelines the Use of Animals in Research of the International Association for the Study of Pain and the directive of the European Parliament (2010/63/EU).

### Inflammatory substances, drugs

We used two different inflammatory agents on the dura mater: CFA contained dried, inactivated *Mycobacterium tuberculosis* in mineral oil (Sigma-Aldrich, St. Louis, MO, USA) and IS contained 1 mM bradykinin, 100 μM prostaglandin, 1 mM serotonin, 1 mM histamine, (pH 5.0) in 10 mM HEPES buffer. Control groups received 0.9% physiological saline or synthetic interstitial fluid (SIF) (135 mM NaCl, 5 mM KCl, 1 mM MgCl_2_, 5 mM CaCl_2_, 10 mM glucose, in 10 mM HEPES buffer, pH 7.3). Lidocaine (20 mg/mL; Egis, Budapest, Hungary) was diluted with physiological saline to have a final concentration of 10 mg/ml (1%).

### Experimental groups

#### Unstimulated animals

Two groups of animals with no surgery/surgical intervention.

#### NAT group

Animals were anesthetized and fixed in a stereotaxic apparatus for the same time period as the animals in 2CFA/2PHYS group (*n* = 3).

#### FRE group

Animals were anesthetized and placed on a heating pad as long as the procedure of the 2CFA or 2PHYS animals was held (*n* = 3).

#### 2PHYS/2CFA; 4PHYS/4CFA groups

Animals underwent surgery, on the dural surface physiological saline (PHYS groups) or CFA (CFA groups) was applied. The survival time was 2 (2PHYS/2CFA) or 4 h (4PHYS/4CFA) after the CFA/saline treatment (*n* = 4, *n* = 4; *n* = 3; *n* = 3).

#### LCFA/LPHYS groups

Rats in these groups underwent the same procedure as 2CFA and 2PHYS groups, but lidocaine was used to anesthetize the scalp before the incision was made on the head (*n* = 2, *n* = 2).

#### 2SIF/2IS; 4SIF/4IS groups

**Fig. 1:**
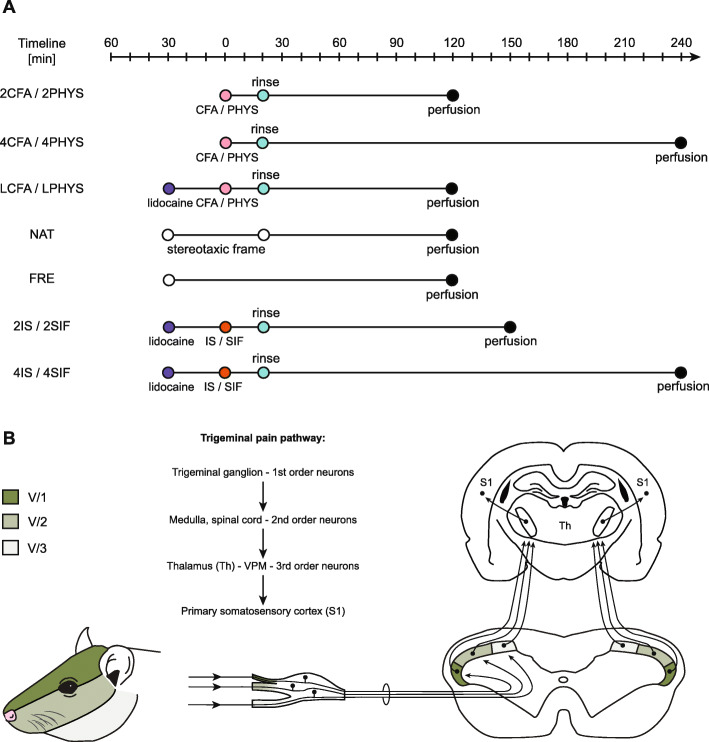
**a**: Schematic timeline of the experimental design. The CFA/saline/IS/SIF treatment was considered as “0” timepoint. LCFA, LPHYS, 2IS, 2SIF, 4IS, 4SIF groups received lidocaine before the skin incision. 20 min after dural treatment, the surface of the dura was rinsed off with either saline or SIF. Animals in NAT and FRE groups were only anesthetized and placed in a stereotaxic frame (NAT) or left on a heating pad (FRE) as long as animals in other groups. **b**: Schematic figure of trigeminal pain pathway. Dermatome distribution of the trigeminal nerve divisions of the face. Trigeminal ganglion contains the first-order neurons, second-order neurons are found in the spinal cord and medulla. Schematic figure of the dorsal horn shows the somatotopic representation of the three branches of the trigeminal nerve. The ventral (darkest), intermediate and dorsal part of the dorsal horn considered to be equivalent to the area of the ophthalmic (V/1), maxillary (V/2), and mandibular (V/3) branch. Figure of the coronal brain section represents the third-order neurons in the thalamus (Th) and neurons in the primary somatosensory cortex (S1)

2.5- or 4-h survival time was chosen to make sure we avoid activation due to the surgery. Animals in these groups had the same surgical procedure as rats in LCFA/LPHYS group, received dural SIF/IS treatment and were perfused after 2.5 h (2SIF/2IS) or 4 h (4IS/4SIF) (*n* = 6/group) (Fig. 1a).

### Procedures

The animals were deeply anesthetized using 4% chloral hydrate (400 mg/kg; intraperitoneal (i.p.)) and placed in a stereotaxic apparatus. The scalp was anesthetized by infiltration of lidocaine (dose of 4.5 mg/kg; subcutaneously) in LCFA, LPHYS, 2SIF, 2IS, 4SIF, 4IS groups. A 2 mm × 2 mm hole (5 mm posterior from the bregma and 3 mm lateral of the midline, above the right hemisphere) was carefully drilled with slow speed, using saline for cooling, avoiding any injury of the dura mater. On the dural surface 10 μL CFA (in groups 2CFA, 4CFA, LCFA) or IS (2IS, 4IS) was applied. Control groups received physiological saline (2PHYS, 4PHYS, LPHYS groups) or SIF (in groups 2SIF, 4SIF). To prevent the local spreading of the chemicals, we adjusted the position of the head in the stereotaxic frame, so the dorsal surface of the skull was horizontal. After 20 min the area was washed with either physiological saline or SIF. To prevent drying out of the dura saline or SIF soaked cotton ball was placed carefully on top. After the surgery animals were kept under anesthesia and placed on a heating pad. The rats were transcardially perfused 2 h (2PHYS, 2CFA, LPHYS, LCFA, NAT, FRE), 2.5 h (2IS, 2SIF) or 4 h (4PHYS, 4CFA, 4IS, 4SIF) after the application of the inflammatory mediators. For the perfusion 50 mL of 0.1 M phosphate-buffered saline (PBS) and 200 mL of 4% phosphate-buffered paraformaldehyde (PFA) was used. The entire brain with the cervical spinal cord was removed, and superficial, angled rostrocaudal cut was made on the ventral, left side of the brainstem and spinal cord. After sectioning this mark allowed us to determine the orientation and the correct rostrocaudal order of the free-floating sections.

### Immunohistochemistry

After postfixation (4% PFA overnight) and cryoprotection with 30% sucrose solution (after gradually increased concentration), cryostat sections of 30 μm thickness were cut. Thirty serials of sections were collected into 10 wells starting from one millimeter rostrally to the obex. Every tenth section was used for staining. For the c-Fos immunohistochemistry free-floating sections were blocked using 0.3% H_2_O_2_ in PBS. After several washes with PBS containing 1% Triton-X-100 (PBS-T), sections were incubated with 10% goat serum in PBS-T for an hour. Incubation with primary antibody for c-Fos (1:2000, sc-52, Santa Cruz Biotechnology, Dallas, TX, USA) was performed on a shaker overnight at room temperature. The reaction was visualized using Vectastatin Elite avidin-biotin kit (PK6101, Vector Laboratories, Burlingame, CA, USA) with 3,3′-diaminobenzidine (Sigma-Aldrich, St. Louis, MO, USA) intensified by nickel-ammonium-sulfate. The specificity of the immune reaction was tested by omitting the primary antiserum. The sections were mounted on glass slides and dried overnight, cleared in xylene, and coverslipped.

### Counting of immunopositive cells

An observer blind to the procedures counted the immunoreactive cells for c-Fos in laminae I-II of the dorsal horn using Nikon Optiphot-2 light microscope (Nikon, Tokyo, Japan) under 10x objective. The cells were also counted according to the somatotopic representation of the trigeminal nerve branches (based on Strassman and Vos, 1993 [13]). The ventral, intermediate, and dorsal parts of the dorsal horn considered to be equivalent to the area of the ophthalmic (V/1), maxillary (V/2), and mandibular (V/3) branch. (Fig. 1b).

Representative photographs were taken by an AxioImager M2 microscope equipped with AxioCam MRc rev.3 camera (Carl Zeiss, Germany) with a 20x objective.

### Statistical analysis

The collected data were analyzed in the 2IS, 2SIF, 4IS, and 4SIF groups. The effects of treatments on c-Fos cell numbers in various distances from the obex between the treated and untreated sides were examined with mixed-design variance of analysis (MIXED-ANOVA) models with distance, treatment, and side as repeated measures (within-subject factor) and group as between-subject factors. *p* < 0.05 was considered statistically significant. Pairwise comparisons were used on estimated marginal means by taking into account the presence or absence of interaction; Holm-Sidak method was performed to adjust *p*-values. For the statistical analysis, IBM SPSS 24.0 (IBM Corp, Armonk, NY, USA) was used. For all other groups, no statistical analysis was used because of the small sample size and to reduce the number of animals used to a minimum. Graphs were made in GraphPad Prism 8.0.1, data are showed as mean + SEM on all figures.

## Results

### Stereotaxic frame

**Fig. 2:**
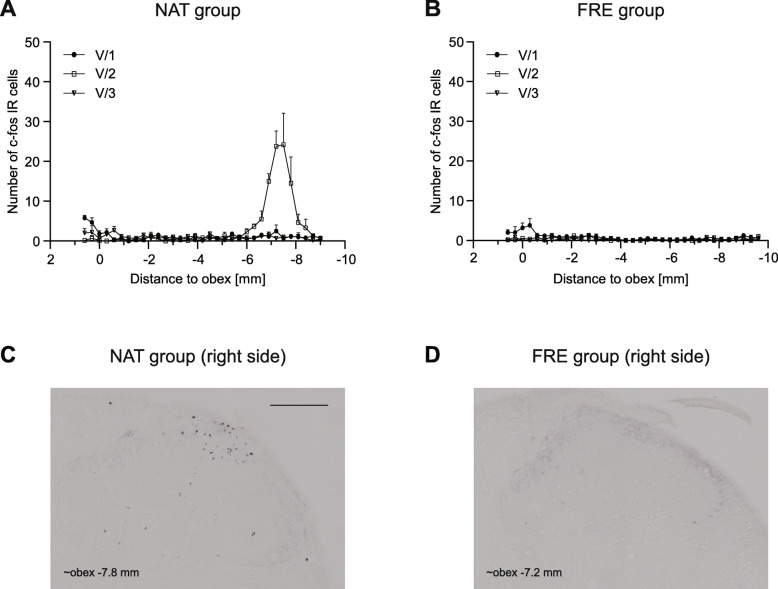
Diagrams show the mean number of c-Fos IR neurons in the dorsal horns in NAT **a** and FRE **b** groups. Representative photomicrographs of the dorsal horn from NAT **c** and FRE groups **d**. Scale bar: 200 μm. Use of stereotaxic frame caused robust increase of c-Fos positive cells in the area corresponding to maxillary nerve in NAT group. Number of c-Fos IR cells was minimal in FRE group (maximal cell number 3.83 ± 1.68 in the ophthalmic nerve area) (mean + SEM)

The application of the stereotaxic frame caused bilateral dense, localized increase in the number of immunoreactive (IR) cells in the somatotopic area of the maxillary nerve (maximum cell number (V/2) 24.17 ± 7.96 at obex − 7.5) (Fig. 2a).

### Anesthetic effect (chloral hydrate) and perfusion

The effect of chloral hydrate and the perfusion in the trigeminal nerve area was negligible (maximum cell number (V/1) 3.83 ± 1.68 at obex − 0.3, Fig. 2b).

### CFA

Dural CFA did not cause noticeable increase in the number of immunoreactive cells compared to physiological saline neither 2 h nor 4 h after administration. There was no difference in the number of cells between the right and left dorsal horns. (Figure not shown.)

**Fig. 3:**
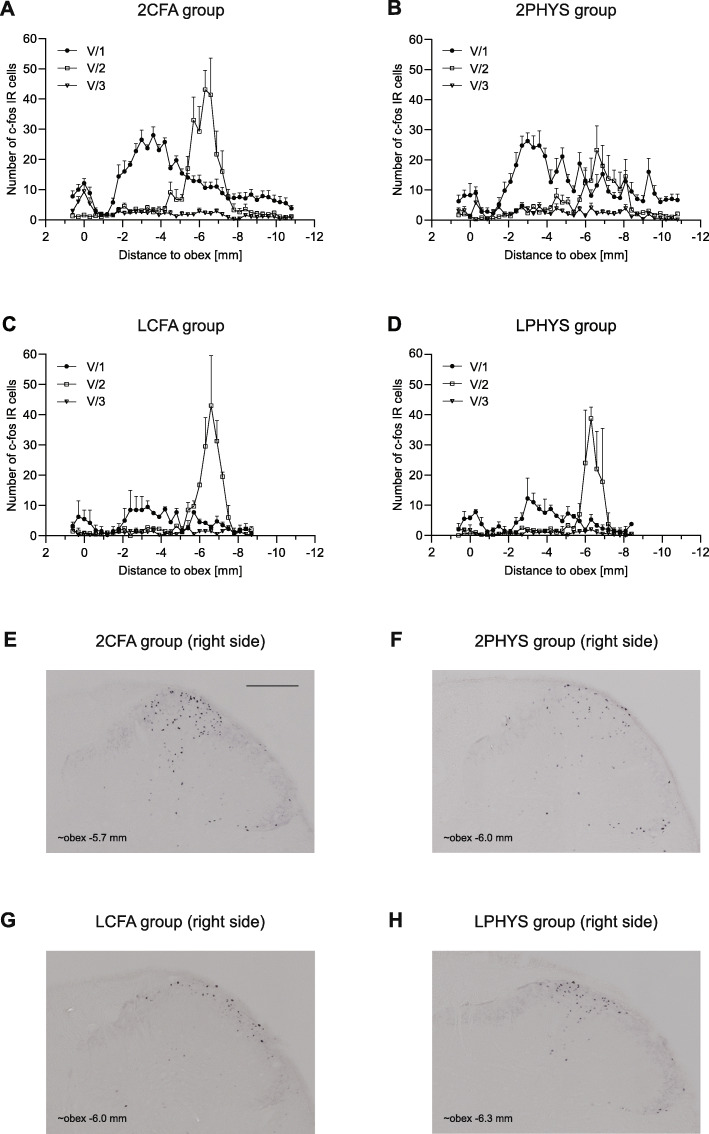
Diagrams illustrate mean number of c-Fos IR neurons in the dorsal horns 2 h after CFA treatment. The number of the cells is displayed according to somatotopic representation of V/1, V/2, V/3 in 2CFA **a** and 2PHYS **b** groups. Diagrams show the mean number of c-Fos IR cells in LCFA **c** and LPHYS **d** groups. Representative photomicrographs from 2CFA **e**, 2PHYS **f**, LCFA **g**, LPHYS **h** group showing the right dorsal horns. Scale bar: 200 μm. Lidocaine decreased the number of c-Fos positive cells compared to 2CFA and 2PHYS group especially in the V/1 area of the dorsal horns (mean + SEM)

However, when the cells were counted based on the trigeminal somatotopy, substantial increase was found in the maxillary nerve area (2CFA, 2PHYS), but no difference was seen between the CFA treated and saline-treated animals (Fig. 3a, b). A slight increase was observed in the ophthalmic nerve area without any difference between the two groups. The labeled cell number was negligible in the mandibular nerve area (Fig. 3a, b).

**Fig. 4:**
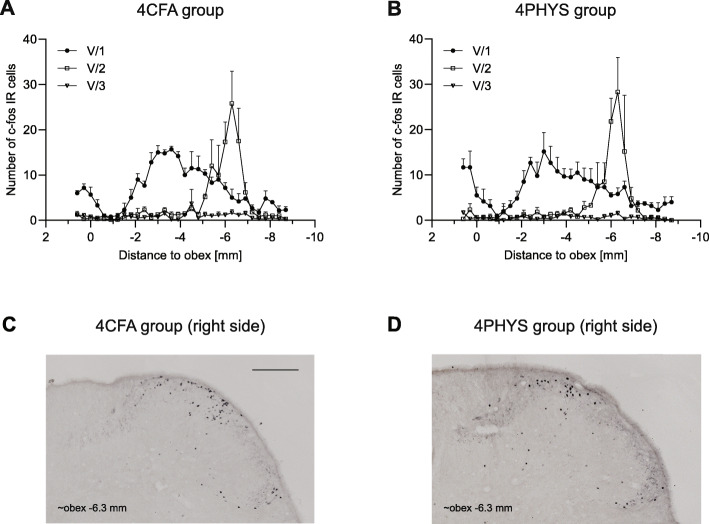
Diagrams illustrate the number of c-Fos positive neurons (mean + SEM) 4 h after CFA **a** or saline **b** treatment. Representative photomicrographs of the dorsal horns in 4CFA **c**, 4PHYS **d**. Scale bar: 200 μm. Cells were displayed according to somatotopic area of V/1, V/2, V/3 nerve. No difference was seen between 4CFA **a** and 4PHYS **b** group

There was no difference between the 4CFA and 4PHYS groups. The number of c-Fos IR cells was less after 4 h compared to the 2 h group (Fig. 4.).

### Lidocaine

Subcutaneous lidocaine applied on the scalp decreased the number of c-Fos immunopositive cells compared to 2CFA group, especially in the area of the ophthalmic branch. Dural application of CFA (Fig. 3c) did not cause robust changes compared to saline in this case either (Fig. 3d).

### IS

**Fig. 5:**
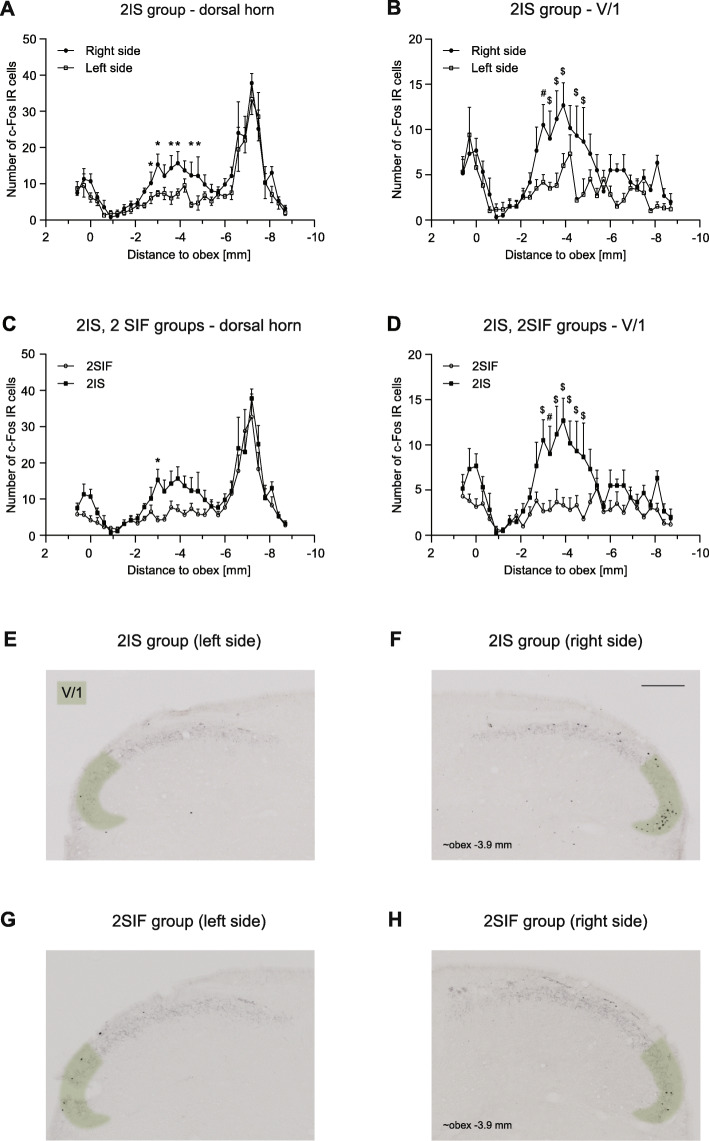
Diagrams showing c-Fos immunoreactive cells across different levels of TNC (mean + SEM) in the whole dorsal horn **a**, **c** and the V/1 area **b**, **d** 2.5 h after IS **a**, **b**, **c**, **d** or SIF **c**, **d**. Representative photomicrographs showing c-Fos IR cells in the dorsal horns of 2IS **e**, **f**, 2SIF groups **g**, **h**. Green highlighting shows the V/1 area in the dorsal horn. Scale bar: 200 μm. We found significant difference between right and left side of the dorsal horn laminae I-II **a**, and the ventral part of the dorsal horn **b**. IS caused significant difference in the number of c-Fos positive cells in the V/1 area **d**. Peak at obex follows the activation pattern of FRE, substantial peak at obex (− 6) - (− 8) mm is consistent with the increased number of cells found in NAT group (*p < 0.05, #*p* < 0.01, $*p* < 0.001)

**Fig. 6:**
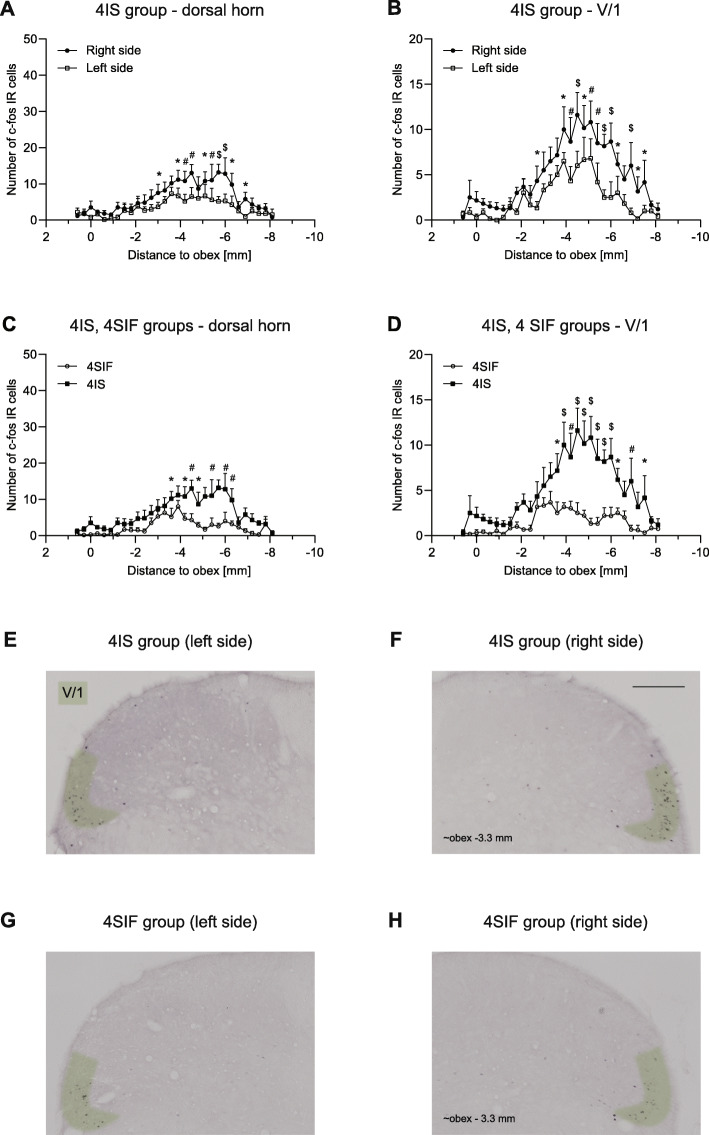
Diagrams illustrate number of c-Fos positive cells in 4IS, 4SIF groups **a**-**d**. Photomicrographs showing c-Fos expression in the dorsal horns of 4IS **e**, **f** and 4SIF groups **g**, **h**. Green highlighting shows the V/1 area in the dorsal horn. Scale bar: 200 μm. IS significantly increases the number of c-Fos IR neurons both in the whole dorsal horn **a** and the V/1 area **b**. IS increased the number of c-Fos positive cells compared to SIF in the whole dorsal horn **c** and in the ophthalmic nerve area **d** (mean + SEM; *p < 0.05, #*p* < 0.01, $*p* < 0.001)

Animals showed significant increase in the number of c-Fos labeled cells in the spinal trigeminal nucleus caudalis both 2.5 h and 4 h after the dural application of IS (Figs. 5 and 6.).

In the 2.5 h group, these changes were more prominent in the somatotopic area of the ophthalmic nerve. Two peaks of activation were found among the rostrocaudal axis. The first peak is at the level of the obex which follows the activation pattern of FRE, while the second was at obex (− 2) - (− 6) mm which in 2IS significantly higher compared to 2SIF. Also, we found significant difference between the right (ipsilateral) and left (contralateral) side at the same rostrocaudal levels. A substantial peak (at obex [− 6] - [− 8] mm) can be seen in the sensory area of the maxillary nerve which is consistent with the peak found in NAT and is not different among 2IS and 2SIF. In the mandibular nerve area, the number of labeled cells was negligible without any difference between the groups. The result of the immunohistochemical staining from the 4SIF and 4IS groups is shown in Fig. 6. The activation pattern is comparable to the peak seen in 2IS group after 2.5 h in the V/1 area. The fact that we saw similar activation pattern in the V/1 area suggests sustained stimulation of the ophthalmic nerve. The substantial increase in number of c-Fos IR cells which was seen in the 2SIF and 2IS groups in V/2 cannot be found in the 4-h survival groups. The number of c-Fos positive cells in the V/3 area is negligible. After 4 h there was significant difference between 4SIF and 4IS group which was present both in the dorsal horn and V/1 area (Fig. 6.).

## Discussion

Fos protein is a widely used marker for locating activated neuronal populations during nociception [14]. C-Fos is an immediate-early gene, its transcription starts within 5 min after the stimulus occurrence [15], and its expression can be already detected at 30 min [16]. Its half-life is 2 h, therefore, it is important to plan the experiment and to establish endpoints according to this time window [17].

Measurement of c-Fos is widely used in headache models, but it is thought to be unspecific since various stimuli might change its expression. In the dural inflammation model, used by our group and many others, surgery (anesthesia, stereotaxic frame – V/1, V/2, skin incision – V/1, etc.) can activate neurons which might interfere with proper data analysis and could be the pitfalls of this method (Fig. 1b).

To better understand these modifying factors and the pattern of the activated cells, we tested the effect of i.p. anesthetic drug, perfusion, stereotaxic frame, and the anesthesia of the skin and scalp by lidocaine. The expression changes were examined in the spinal trigeminal nucleus caudalis, and the rostrocaudal and somatotopic distribution was also taken into account to increase specificity.

It is known for a long time that urethane can cause activation in the trigeminal system even without any facial stimulation. Strassman and Vos found that not just urethane, but immediate perfusion in pentobarbital anesthesia causes substantial activation in the caudal medulla [13]. Based on this study, we decided to examine the effect of chloral-hydrate anesthesia and perfusion (FRE group). The number of c-Fos IR cells mildly increased in the V/1 area, but no activation was found in the area of V/2 and V/3. The rostrocaudal distribution and the number of c-Fos IR cells were comparable to the previous findings of Strassman and Vos [13].

A stereotaxic frame is essential in cranial surgeries to completely stabilize the head and ensure experimental procedure consistency of each employed animal, but in our experiment, it caused a robust and highly variable activation in the area of the maxillary nerve (NAT group). Animals were held in the frame using three points; ear bars placed in the ear canals and snout were fixed by the front incisors. Innervation of the upper frontal incisors is provided by the maxillary nerve, which can be responsible for the activation effect seen in the V/2 area [18]. External ear canal is innervated by auriculotemporal nerve (from V/3), greater and lesser auricular nerve (from C2, C3 spinal nerves), Arnold’s nerve (from vagus, glossopharyngeal and facial nerve) [19]*.* As we did not witness any increase in the number of activated cells in the V/3 area, the aforesaid increase in V/2 might be mainly due to the fixation of the snout. The substantial activation found in V/1 area is consistent with the changes seen in FRE group that underwent only anesthesia and perfusion.

In addition, for a more specific analysis of the effects of chemical stimulation of the dura, lidocaine was applied subcutaneously on the scalp (LCFA, LPHYS groups), before the surgical incision and CFA treatment, which decreased the number of c-Fos IR cells in the area of ophthalmic nerve compared to 2CFA and 2PHYS group. Recent tracing studies suggest that intracranial and extracranial structures share afferent fibers, which innervate the dura mater and after penetrating the calvarium they supply the periosteum [20, 21]. Surgical incision of the skin can activate these neurons and can hide the effect of the chemical stimulation of the dura, thus the use of lidocaine is essential when examining dural nociception. The distribution of c-Fos in V/2 area was similar to the NAT group’s activation pattern and based on the rostrocaudal appearance and variance, we can assume the effect of the stereotaxic frame here as well. These findings might help to identify the effects of various experimental procedures in the future to make data analysis easier.

In this study, we applied two different inflammatory agents on the dura mater, namely CFA and IS. Sub- or intradermal injection of CFA is commonly used in inflammatory and neuropathic pain models: when injected into hind paw, it caused hyperalgesia and edema with rapid onset [22]. While the first effects develop in few hours and peak after 24–72 h, studies also described long-term complications, such as granulomatous inflammation, skin ulceration, focal necrosis [23]. We chose 2-h and 4-h survival time for CFA groups to avoid these long-term effects of the treatment. In our experiments, CFA applied over the right hemisphere, with 2-h survival time, did not cause any difference in the number of c-Fos positive cells between the right and left dorsal horns of the spinal trigeminal nucleus. Moreover, no changes were detected in the number of immunoreactive cells in the CFA treated and saline group either. When lidocaine was used to anesthetize the skin (LCFA and LPHYS groups) before CFA or PHYS treatment, the same outcome was seen, no difference was found among these groups. Even with the longer, 4-h survival time, we could not observe any alterations between the 4CFA and 4PHYS groups either.

Previous electrophysiological studies showed that when inflammatory agents are used on the dural surface, secondary sensitization occurred after the delay of 2–3 h in the spinal trigeminal nucleus [5, 24]. Adjuvants like CFA are frequently used for polyclonal antibody production in animals; thus, they contain substances which slow down the breakdown of Mycobacteria, prolonging its effect [23]. Other studies’ results also support the delayed effect of CFA, e.g., its subcutaneous injection can induce swelling and hyperalgesia with peaks around 24 h [22]. In a model of parotitis, CFA was injected into the parotid gland, and increased c-Fos expression was detected in the spinal trigeminal nucleus after 2, 24, 72 h, which peaked at 72 h [25]. Moreover, it is important to emphasize that CFA was applied permanently in these experiments (subcutaneous, intracutaneous application, or injected into the parotid gland), while we rinsed the surface of the dura 20 min after application, which might mitigate its short term effects. The number of studies investigating dural treatment of CFA is limited; Lukács et al. applied CFA on the surface of the dura and rinsed the surface after 20 min, and they described signs of inflammation after 4 and 24 h in the trigeminal ganglion; calcitonin-gene related peptide (CGRP), interleukin-1β levels were increased compared to saline-treated group [4]. With longer survival time (seven days), they detected increased number of c-Fos positive cells in the spinal trigeminal nucleus [26]. Based on the studies mentioned above, we can assume that the variances between the site and the mode of application might induce different effects (onset and duration), consequently, in different experiments, various neuronal activation and sensitization patterns were described; the first effects developed over 2, 4 h or more, and in most cases, the peak was between 24 and 72 h. This is consistent with the time course of delayed-type hypersensitivity reaction, which could be the main mechanism of action of the CFA application and can explain the demonstrated variations [27]. In the inflammation pain models, the effects of inflammatory mediators are examined in the neurons of the dorsal root ganglia that are part of the spinothalamic pathway [28], and information processing might be slightly different in the trigeminal system. Hoffman and Matthews found that the proportion of the sympathetic fibers is higher in spinal nerves than trigeminal nerves [29]. The function of the sympathetic nervous system (SNS) in pain processing is not fully understood: it can play a role in the descending inhibition of pain [30], but on the other hand, studies showed that catecholamines might be able to sensitize nociceptors [31]. There is also a link between SNS and immune system; primary and secondary lymphoid organs receive sympathetic innervation [32], and immune cells express adrenergic receptors [33, 34]. In inflammation, immune cells upregulate alpha-1 receptors which can stimulate release of proinflammatory cytokines [35, 36]. The higher proportion of the sympathetic fibers in spinal nerves might be able to stimulate more immune cells which, probably could initiate inflammation faster. The main mechanism of action of the CFA is cell-mediated, this could make it more sensitive to the variances in the autonomic nervous system compared to other inflammatory agents [37]. These phenomena might contribute to our results not showing the activation of the trigeminal system with shorter survival times.

Edvinsson et al. suggested that sustained trigeminal activation produced by dural or temporomandibular CFA administration could model the transformation from episodic to chronic migraine [38]. Nociceptor activation causes release of neuronal mediators like CGRP, which initiate a local inflammatory response [39] and possibly induce continuous activation and sensitization of the trigeminal system [40], creating a self-amplifying process [41]. This can be paralleled by the effect of repeated migraine attacks, which might generate sustained neurogenic inflammation or so-called neurogenic neuroinflammation [38]. As the dural or temporomandibular administration of CFA can cause prolonged activation of the trigeminal system with continuous inflammation [4, 42], it might be able to mimic the chronification process. In our experimental design, it was important to avoid any destruction or injury on the dura mater; thus, we applied CFA for 20 min and sacrificed the animals after a delay of 2 and 4 h to avoid any long term complications of the application, such as granulomatous infection and focal necrosis, which would appear after a longer delay. It is possible that the time of application or the survival time was too short to activate the secondary sensory neurons in the spinal trigeminal nucleus. As CFA did not cause increase in the number of c-Fos IR cells in the trigeminal system within this time frame, its dural application might be more useful in the investigation of a trigeminal activation with a longer delay.

Sterile inflammation (neurogenic inflammation) can play a role in the pathomechanism of migraine [43], and application of IS on the dura mater is widely used to mimic this process in animals [44, 45]. IS contains inflammatory agents; bradykinin, prostaglandin E_2_, histamine, and serotonin, causing activation of the neurons directly and indirectly through release of other mediators. Activation and sensitization markers can be detected as soon as 20 min after the stimulation in the first-order neurons, and after 2 h in the second-order neurons [5, 46]. We examined the number of c-Fos immunoreactive cells after dural IS in spinal trigeminal nucleus caudalis. Previous mapping studies helped us to learn more about the innervation of the dura mater and the somatotopic representation of the trigeminal nerve [7, 47]. These studies allowed us to be the first to present the effect of chemical stimulation in detail. Compared to SIF, IS caused significant increase in the number of labeled cells was the most prominent in the somatotopic area of the ophthalmic nerve, which is responsible for the somatosensory innervation of the dura mater [48]. The innervation of the supratentorial dura is mostly ipsilateral [48], consequently, we found significant difference between the right (ipsilateral) and left (contralateral) caudal spinal trigeminal nucleus. In several studies, a catheter was placed into the cisterna magna or over the parietal hemisphere [49, 50]. If the mediators are injected to the cisterna magna, side differences cannot be seen because this area has bilateral innervation and the substance spreads around the cisterna [51]. The significant difference in the number of c-Fos IR cells between the IS and SIF groups persists even after 4 h. From earlier studies, it is known that IS can cause activation and sensitization of the trigeminal system: it has been demonstrated that chemical stimulation of the dura caused hyperresponsiveness and increased the receptive field of the TNC neurons [52], and Edelmayer et al. presented that dural IS was able to induce cutaneous allodynia not only in the trigeminal dermatome but also in the hind-paws. In their experiments, withdrawal threshold to von Frey filament stimulation decreased slowly after dural IS application, reached its maximum after 3 h, and returned to baseline after 5–6 h [53]. These findings suggest that the central sensitization and allodynia is an indirect effect of IS and can explain the persistent activation found throughout our experimental time points.

Application of IS on the dura mater triggers activation and sensitization of the trigeminal system, showing several similarities with migraine. Allodynia is present in ~ 63% of the patients, in the animal model the time course of the central sensitization is consistent with the cutaneous allodynia seen in patients [54, 55]. Many studies demonstrated that IS evoked activation respond to migraine-specific and non-specific drugs, which supports that the chemical stimulation model has a place in drug development [52, 56]. Though dural application of IS cannot demonstrate all aspects of the disease, based on these findings it can be a valid animal model of migraine.

Our data support that dural application of IS is a relevant animal model of migraine, however, application of CFA can be an alternative method if the longer delay of the latter is taken into account as they recruit leucocytes, which release inflammatory mediators instead of direct neuronal activation.

## Conclusion

Counting cells according to the somatotopic organization in the trigeminal system helps the data analysis, chemical stimulation over the parietal lobe has the most prominent effect in the ophthalmic nerve area. Based on other’s and our results, somatotopic representation of the scalp and the parietal dura mater overlaps; thus, application of lidocaine is essential to examine the effect of chemical stimulus in a more specific way. In summary, IS applied on the dura mater can serve as an approach to model acute migraine, while dural application of CFA might be used as a long-term trigeminal activation model.

## Data Availability

The datasets used and/or analyzed during the current study are available from the corresponding author on reasonable request.

## Abbreviations

CFA: Complete Freund’s Adjuvant
2CFA/4CFA: Complete Freund’s Adjuvant treated groups
CGRP: Calcitonin-gene related peptide
i.p.: Intraperitoneal
IR: Immunoreactive
IS: Inflammatory soup
2IS/4IS: Inflammatory soup treated groups
PBS: Phosphate buffered saline
PBS-T: PBS containing 1% Triton-X-100
PFA: Paraformaldehyde
2PHYS/4PHYS: Physiologic saline treated groups
SIF: Synthetic interstitial fluid
2SIF/4SIF: Synthetic interstitial fluid treated groups
SNS: Sympathetic nervous system
V/1: Ophthalmic nerve
V/2: Maxillary nerve
V/3: Mandibular nerve
